# Insufficient Bone Mineralization to Sustain Mechanical Load of Weight in Obese Boys: A Cross-Sectional Study

**DOI:** 10.1210/clinem/dgad760

**Published:** 2024-01-02

**Authors:** Fabienne Emeriau, Jessica Amsellem-Jager, Natacha Bouhours-Nouet, Aurelie Donzeau, Stephanie Rouleau, Solène Rerat, Emmanuelle Labarre, Lucie Levaillant, Régis Coutant

**Affiliations:** Department of Pediatric Endocrinology, University Hospital, 49000 Angers, France; Department of Pediatric Endocrinology, University Hospital, 49000 Angers, France; Reference Center for Rare Pituitary Diseases, University Hospital, 49000 Angers, France; Specialized Center for Obesity, University Hospital, 49000 Angers, France; Department of Pediatric Endocrinology, University Hospital, 49000 Angers, France; Reference Center for Rare Pituitary Diseases, University Hospital, 49000 Angers, France; Specialized Center for Obesity, University Hospital, 49000 Angers, France; Department of Pediatric Endocrinology, University Hospital, 49000 Angers, France; Department of Pediatric Endocrinology, University Hospital, 49000 Angers, France; Department of Pediatric Endocrinology, University Hospital, 49000 Angers, France; Department of Pediatric Endocrinology, University Hospital, 49000 Angers, France; Department of Pediatric Endocrinology, University Hospital, 49000 Angers, France; Department of Pediatric Endocrinology, University Hospital, 49000 Angers, France; Reference Center for Rare Pituitary Diseases, University Hospital, 49000 Angers, France; Specialized Center for Obesity, University Hospital, 49000 Angers, France

**Keywords:** obese, male, bone mineral content, testosterone, insulin, fat mass

## Abstract

**Context:**

The increase in bone mineral content (BMC) and density (BMD) measured by dual-energy x-ray absorptiometry (DXA) in obese children may not sustain the mechanical load associated with weight, and the factors influencing bone mineralization are not well known.

**Objective:**

We described bone mineralization in boys with overweight/obesity and leanness in relation to body composition.

**Methods:**

Cross-sectional study in the Pediatric Endocrinology Unit of Angers University Hospital with 249 overweight/obese boys aged 8-18 who underwent DXA and insulin, testosterone, and IGF-1 measurements. Bone mineralization was compared with data from 301 lean boys of similar age and height from NHANES 2011-2015, using the same DXA model. Path analyses were performed to evaluate factors associated with total body less head (TBLH) BMC.

**Results:**

The mean age- and height-adjusted difference in TBLH BMC between obese and lean boys was 241 ± 20 g/cm^2^. Each 1 kg/m^2^ increase in BMI was associated with +39 ± 6 g of TBLH BMC in lean subjects vs + 25 ± 3 g in obese subjects (*P* < .05). Each 1 kg/m^2^ increase in lean BMI (LBMI) was associated with +78 ± 5 g of TBLH BMC in lean and obese boys, and each 1 kg/m^2^ increase in fat mass index (FMI) was associated with a decrease of 9 ± 3 g of TBLH BMC. The TBLH BMC was directly positively influenced by LBMI and indirectly and positively influenced by IGF-1, testosterone, and insulin (mediated through height and LBMI). FMI indirectly influenced TBLH BMC, both positively through LBMI and negatively through its negative impact on IGF-1 and testosterone.

**Conclusion:**

The increase in bone mineralization in obese children does not adapt to the increase in body mass.

According to the World Health Organization, 18% of youth aged 5-19 worldwide were overweight or obese in 2016 (1). The consequences of obesity on bone health in children are multiple and somewhat contradictory. Children with obesity have a 25% higher risk of extremity fracture than children of average weight (2-4). They are less likely than lean children to engage in strenuous physical activities, a significant determinant of bone health (5). In contrast, those who engage are more prone to fall due to an abnormal gait associated with obesity (6). Regarding bone health measurements, obese children have a higher areal bone mineral density (BMD) and bone mineral content (BMC) compared to children with an average weight, with a more marked difference in girls than in boys (7). However, increased height during childhood in children with obesity compared to lean children may be a confounding factor for areal BMC and BMD measurements (8, 9), whereas most studies did not adjust for height (7).

For studying bone mineralization, dual-energy x-ray absorptiometry (DXA) is the preferred method in children: the posterior-anterior lumbar spine (L1-L4, fast array) areal BMD (lumbar BMD) and the total body less head areal BMC (TBLH BMC) are considered the best estimates of bone mineralization in children (9). DXA can also be used to measure lean and fat mass (9). Areal bone mineral content and density rely on the two-dimensional areal projection of a three-dimensional object (bone). They are intrinsically related to the size of the bone, which is inherently related to the child's height. Thus, DXA overestimates bone density and content of a tall child and underestimates bone density in a short child. Several mathematical and statistical size adjustment techniques have been developed to account for the size limitation of DXA (9, 10). However, there is currently no consensus as to the best method of adjustment. In addition, obesity may be associated with altered trabecular and cortical bone microarchitecture in adults and children, as measured by high-resolution peripheral quantitative computed tomography (11, 12). This raises the question of whether the apparent increase in BMD is sufficient to support the increased mechanical load in obese children (7, 13).

In lean, healthy children, there is a correlation between BMC and lean body mass, suggesting that the amount of lean mass determines bone mineralization (14). Twin studies have confirmed that lean mass, not fat mass, is the primary determinant of BMC (15). The heritability of bone and lean body mass is estimated to be 60% to 85% and 50% to 70%, respectively (16, 17). Besides genetic factors, studies in children and adolescents have shown that physical activity and calcium intake are also related to bone health (18). Hormonal factors, such as growth hormone, insulin-like growth factor-1 (IGF-1), sex steroids, thyroid hormones, and insulin interact with each other, modulate the differentiation and activity of osteoblasts, osteoclasts, muscle, and fat cells, and affect bone health, adipose tissue, and muscle mass (19). Growth hormone, insulin, IGF-1, and testosterone promote the development of muscle mass; growth hormone, thyroid hormones, and testosterone are lipolytic; and insulin and IGF-1 promote the development of fat mass (20). Obesity also modulates these hormonal factors: obese children and adolescents have lower levels of growth hormone and testosterone (in boys) than lean subjects, higher levels of insulin, thyrotropin (thyroid stimulating hormone [TSH]) and free triiodothyronine (T3), and variable levels of IGF-1 (21-24). Whether these hormones are related to bone mineralization in subjects with obesity and whether they act differently in boys and girls is unknown.

The first objective of this cross-sectional study was to describe bone mineralization in a group of 249 obese and overweight boys aged 8 to 18 years, according to age and pubertal stage. They were compared to 301 lean (non-overweight, nonobese) boys aged 8 to 18 years matched for height from the United States National Health and Nutrition Examination Survey (NHANES) 2011 to 2015, which used the same DXA device (Hologic Model A densitometer) (25). The subjects were matched for height to overcome the influence of size on TBLH BMC and lumbar BMD. Subjects only included boys because gender differences in bone mineralization, as shown in several studies, may have confounding effects on the results (7). The second objective was to assess whether bone mineralization in children with obesity adjusted to the increased mechanical weight load. The last objective was determining which factors influenced bone mineralization among body composition (lean mass, fat mass) and hormonal factors (insulin, IGF-1, testosterone, free thyroxine [T4], TSH).

## Methods

**Figure 1.**
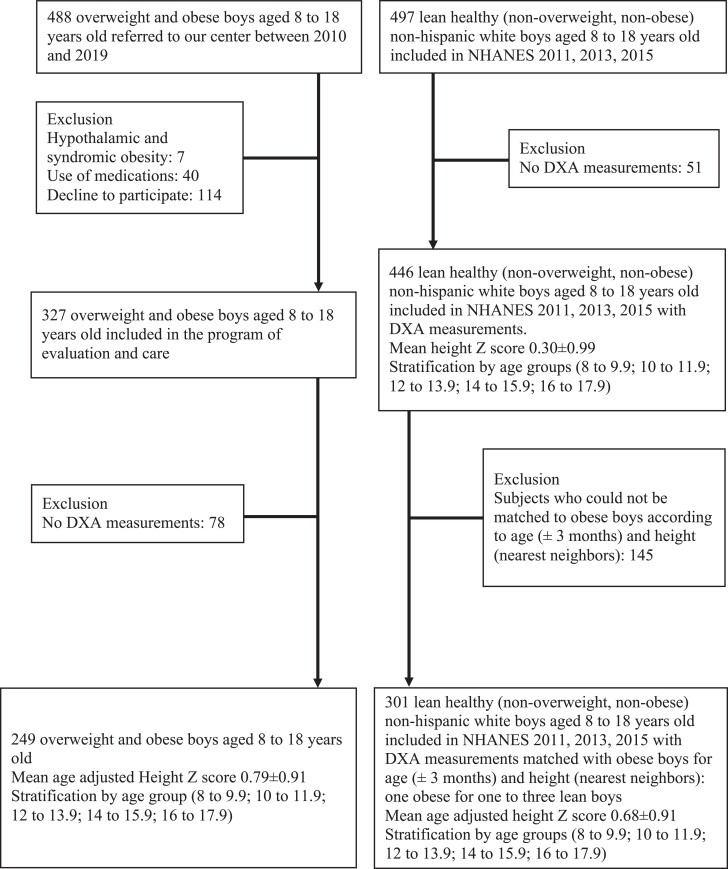
Flowchart of the study.

From 2010 to 2019, this cross-sectional study included obese male children and adolescents aged 8-18 referred to the Pediatric Endocrinology Unit of Angers University Hospital for obesity management. Exclusion criteria were syndromic, genetic, hypothalamic, or endocrine obesity, diabetes, and the use of any medication influencing weight gain. During the recruitment period, 488 boys aged 8-18 were referred to our center. Seven were excluded because of syndromic obesity or obesity of hypothalamic origin (mean body mass index [BMI] and BMI Z-score 32.3 ± 5.0 kg/m^2^ and 2.2 ± 0.4), 40 were excluded because of the use of medications (mean BMI and BMI Z-score 36.5 ± 8.7 kg/m^2^ and 2.5 ± 0.4), 114 declined to participate in the program (no BMI data collected for regulatory reasons), and 78 were excluded because of missing DXA data (mean BMI and BMI Z-score 31.8 ± 6.1 and 2.3 ± 0.3): 249 patients were finally included, 244 of them of White and 5 Black ethnic background (Fig. 1). The care program for subjects with obesity included a comprehensive baseline assessment with metabolic, hormonal, and body composition measurements, and follow-up with lifestyle intervention, group sessions, and appointments with doctors, nurses, dieticians, and psychologists, with meetings every 2 months for 2 years. It received approval by the regional health agency (ARS des Pays de la Loire; ARS-PDL/DEO/CPS/2015/25 DECISION) on behalf of the national health system and all parents signed a written consent (all the children gave their assent, and those old enough to sign the consent form did so) to participate in the program. The retrospective collection of medical data from the patients’ medical records was approved by the ethics committee of Angers University Hospital (Avis 2020/92).

The control boys come from the NHANES 2011 to 2015 studies (25): 497 lean healthy (non-overweight, nonobese) non-Hispanic White boys aged 8 to 18 years old were included in NHANES 2011, 2013, 2015, of whom 51 were excluded because they had no DXA measurements. Matching of controls with obese boys was performed based on age (±3 months) and height (nearest neighbors): 1 obese for 1 to 3 lean boys. Finally, 301 lean, healthy control boys were included.

### Study Design

Height and weight measurements were performed on all patients. Height Z-scores were calculated according to Centers for Disease Control (CDC) references because these references are close to the recently published French references and allowed for the calculation of height-adjusted BMC and BMD Z-scores (see below) (26-28). Physical examinations included blood pressure measurement (mmHg) and, for obese boys only, pubertal staging, according to Tanner (29). We calculated BMI for each patient, with the formula: weight (kg)/height (m)^2^, expressed in Z-score relative to age-based CDC references (26). Overweight was defined as a BMI > +1 (corresponding to the 85th percentile) and < +2 Z-score, and obesity as a BMI > +2 Z-score. Calcium intake and physical activity were not collected with a validated questionnaire.

### Blood Analyses

After overnight fasting, blood samples were obtained for each patient and included insulin, total cholesterol, high-density lipoprotein (HDL) cholesterol, triglyceride, and testosterone levels. For overweight and obese subjects, TSH, free T4, and IGF-1 were measured. For lean boys from the NHANES studies, triglyceride levels were not measured before age 10, insulin levels were not measured before age 12, and TSH, free T4, and IGF-1 were not measured. Too many 25-hydroxyvitamin D levels were missing for confident use of the available data.

For obese subjects, lipids were measured by commercially available kits (Advia Chemistry Xpt, Siemens, Munich, Germany), thyroid hormones by immunochemiluminescence (Centaur Siemens, Munich, Germany), plasma insulin was measured by radioimmunoassay (Schering AG Cis Bio International, Gif sur Yvette, France) (CISBIO cat. no. 62IN1PEG, RRID:AB_2890910; https://antibodyregistry.org/search.php?q=AB_2890910), testosterone was measured by radioimmunoassay, after a steroid extraction step, using Orion reagents (Cis Bio International, Gif sur Yvette, France) (Abcam cat. no. ab33863, RRID:AB_778304; https://antibodyregistry.org/search?q=RRID:AB_778304), and IGF-1 by immunoradiometric assay (Beckman Coulter, Villepinte, France). For lean boys from the NHANES studies, biological measurements were performed according to NHANES specifications and analytic guidelines, and assays were different from those used in overweight and obese subjects (30).

### Evaluation of Body Composition

Body composition was investigated by DXA using a Hologic Model A densitometer (Hologic, Waltham, MA, USA) using software version Apex 3.2 in overweight, obese, and lean boys. We analyzed TBLH BMC (g) and lumbar BMD (g/cm^2^). We also calculated the Z-scores, adjusted with height (HAZ), age, gender, and non-Black race (28). Fat and lean mass were also measured by DXA. The FMI (fat mass index) (total body fat mass divided by height squared) and LBMI (lean body mass index) (total lean mass divided by height squared) were calculated and converted in Z-score calculated with fat and lean BMI reference curves in children and adolescents (31).

### Statistical Methods

Continuous variables were expressed as mean ± SD or median (5th and 95th percentiles). Discrete variables were expressed as percentages. Kruskal–Wallis, Wilcoxon rank-sum tests, chi-squared, Student *t* test, and ANOVA were used for comparisons, depending on the normal or non-normal distribution of the variables. Significance was defined as *P* < .05. The first outcomes of interest were TBLH BMC and lumbar BMD. Insulin, total cholesterol, IGF-1, testosterone, TSH, and free T4 were also expressed as Z-score values according to references for age and gender in children (22, 23, 32, 33, local normative data for IGF-1). This allowed us to study age-adjusted relationships between variables.

The quantitative influence of BMI, FMI, and LBMI on bone mineralization (TBLH BMC and lumbar BMD) were studied by multiple regression analyses: age and height were adjusting variables, BMI, or FMI and LBMI and their interactions with the obesity status were independent variables. This way of proceeding allowed us to evaluate whether a 1-unit gain in either BMI, LBMI, or FMI had a similar influence on bone mineralization in lean boys (where this relationship likely reflects the standard response of mineralization to weight) and obese boys.

Finally, to examine the associations of hormones (insulin, IGF-1, testosterone, free T4, TSH) and body composition variables (height, FMI, and LBMI) with the outcome variable (TBLH BMC Z-score), path analyses were conducted using structural equation modeling framework: this allowed to investigate patterns of effects within the selected system of variables. Simple correlations between variables were performed. Significantly correlated variables were entered into the path analyses. We used the maximum likelihood (ML) estimator. Cutoff criteria for Fit indexes were a nonsignificant χ² *P* value of the model (statistically significant values allow rejecting the model since they indicate a discrepancy between the estimated and observed matrices), a ratio of χ² to df < 3, a comparative Fit index >0.95, a root mean square error of approximation (RMSEA) < 0.08, and a standardized root mean squared residual (SRMR) < 0.05 (34).

We used SPSS Statistics v25 (IBM Corp., Armonk, NY) and Jamovi software version 2.3.18.0 (The Jamovi Project, 2022; https://www.jamovi.org).

## Results

Between January 2010 and January 2019, we included 249 male patients aged 8 to 18. Fourteen were overweight, and 235 were obese. Twenty-one (8%) had reported a history of fractures (≥1 fracture), and this was negatively correlated with lumbar BMD HAZ-score (*r* = −0.145, *P* < .05), but not with TBLH BMC HAZ-score (*r* = −0.05, *P* = .25). They were compared with 301 lean boys from NHANES 2011-2015 matched for height (Fig. 1).

### Clinical, Body Composition, Metabolic, and Hormonal Characteristics of the Boys According to Age and Pubertal Stage

The characteristics of the obese and lean boys according to age groups are reported in Tables 1 and 2. There was a negative correlation between height Z-score and age (*r* = −0.32, *P* < .001).

**Table 1.** Clinical characteristics, body composition, and bone mineralization of overweight/obese and lean boys according to age groups

	8 to <10 years		10 to <12 years		12 to <14 years		14 to <16 years		16 to <18 years	
	Obese	Lean	Obese	Lean	Obese	Lean	Obese	Lean	Obese	Lean
N	33	37	47	47	86	70	51	76	32	71
Age, y	9.1 ± 0.6	9.1 ± 0.6	11.0 ± 0.6	11.0 ± 0.6	13.0 ± 0.6	13.0 ± 0.5	14.9 ± 0.6	14.9 ± 0.6	16.8 ± 0.6	16.9 ± 0.7
Height, m	1.41 ± 0.05	1.40 ± 0.04	1.52 ± 0.07	1.51 ± 0.06	1.62 ± 0.08	1.62 ± 0.08	1.73 ± 0.06	1.72 ± 0.07	1.77 ± 0.08	1.76 ± 0.07
Height Z-score	1.21 ± 0.89	1.03 ± 0.52	1.18 ± 0.99	0.99 ± 0.61	0.84 ± 1.10	0.79 ± 0.86	0.50 ± 0.81	0.51 ± 0.82	0.24 ± 1.09	0.24 ± 1.09
Tanner stage	1 (1; 1)		1 (1; 3)		2 (1; 4)		4 (2; 5)		4 (2; 5)	
BMI, kg/m^2^	26.1 ± 4.1	16.6 ± 1.2	28.7 ± 4.0	17.4 ± 1.5	31.0 ± 4.0	18.8 ± 1.7	35.2 ± 5.2	20.0 ± 1.6	37.6 ± 6.8	20.9 ± 1.9
BMI Z-score	2.20 ± 0.27	0.09 ± 0.68	2.18 ± 0.27	−0.04 ± 0.70	2.19 ± 0.30	0.05 ± 0.68	2.37 ± 0.36	0.00 ± 0.65	2.50 ± 0.39	−0.16 ± 0.71
FMI, kg/m^2^	11.25 ± 2.78	4.34 ± 0.85	12.80 ± 2.75	4.52 ± 1.14	13.31 ± 3.01	4.43 ± 1.15	14.50 ± 3.51	4.21 ± 1.09	14.78 ± 4.51	4.05 ± 0.86
FMI Z-score	1.5 ± 0.27	−0.26 ± 0.50	1.66 ± 0.26	−0.28 ± 0.66	1.66 ± 0.27	−0.36 ± 0.62	1.75 ± 0.31	−0.48 ± 0.63	1.75 ± 0.39	−0.54 ± 0.52
LBMI, kg/m^2^	14.45 ± 1.51	11.77 ± 0.68	15.04 ± 1.67	12.4 ± 0.88	16.54 ± 1.73	14.1 ± 1.40	19.74 ± 2.57	15.31 ± 1.14	21.23 ± 3.35	16.11 ± 1.44
LBMI Z-score	1.15 ± 0.73	−0.53 ± 0.54	1.10 ± 0.77	−0.46 ± 0.55	1.10 ± 0.67	−0.004 ± 0.71	1.42 ± 0.75	−0.19 ± 0.55	1.32 ± 0.99	−0.52 ± 0.71
TBLH BMC, g	897 ± 164	739 ± 81	1147 ± 206	939 ± 142	1489 ± 319	1272 ± 305	1959 ± 392	1590 ± 258	2335 ± 390	1866 ± 327
TBLH BMC Z-score	2.15 ± 1.08	0.66 ± 0.87	2.12 ± 1.19	0.56 ± 0.80	1.67 ± 1.14	0.67 ± 1.30	1.22 ± 1.20	0.06 ± 0.96	1.09 ± 1.10	−0.35 ± 1.06
TBLH BMC-HA Z	1.12 ± 0.75	−0.21 ± 0.83	1.14 ± 0.74	−0.27 ± 0.79	0.51 ± 0.69	0.11 ± 1.04	0.92 ± 0.96	−0.03 ± 0.87	0.81 ± 1.15	−0.33 ± 0.85
Lumbar BMD, g/cm^2^	0.646 ± 0.083	0.657 ± 0.061	0.719 ± 0.087	0.701 ± 0.062	0.822 ± 0.138	0.790 ± 0.129	0.949 ± 0.154	0.904 ± 0.123	1.103 ± 0.106	1.002 ± 0.124
Lumbar BMD Z-score	1.35 ± 0.79	1.20 ± 1.12	1.46 ± 1.10	1.15 ± 0.79	1.46 ± 1.36	1.13 ± 1.25	1.08 ± 1.35	0.66 ± 1.04	1.15 ± 0.82	0.29 ± 0.98
Lumbar BMD HAZ	0.65 ± 1.03	0.88 ± 0.78	0.93 ± 0.96	0.67 ± 0.81	0.66 ± 1.16	0.76 ± 1.12	0.81 ± 1.15	0.45 ± 1.08	0.19 ± 0.97	0.96 ± 0.82
Percent fat mass	42 ± 4	26 ± 4	44 ± 5	26 ± 5	43 ± 5	23 ± 5	41 ± 6	21 ± 4	40 ± 5	19 ± 3

Variables were expressed as mean ± SD or median (5th and 95th percentiles) when non-normally distributed.

Abbreviations: BMC, bone mineral content; BMD, bone mineral density; BMI, body mass index; FMI, fat mass index; LBMI, lean body mass index; TBLH, total body less head; TBLH BMC-HA Z, total body less head bone mineral content height-adjusted Z-score.

**Table 2.** Biological characteristics of overweight/obese and lean boys according to age groups

	8 to <10 years		10 to <12 years		12 to <14 years		14 to <16 years		16 to <18 years	
	Obese	Lean	Obese	Lean	Obese	Lean	Obese	Lean	Obese	Lean
Insulin pmol/L	69 (36; 167)		98 (43; 288)		111 (60; 264)	8 (5; 14)	123 (53; 360)	10 (5; 28)	153 (56; 505)	8 (4; 21)
Insulin Z-score	2.42 ± 1.89		1.94 ± 1.79		1.83 ± 1.38	.0.24 ± 0.88	2.46 ± 2.01	0.39 ± 1.14	3.14 ± 2.24	0.27 ± 1.60
Cholesterol mmol/L	4.2 (2.8; 10.9)	3.7 (2.9; 4.7)	4.3 (3.0; 6.0)	3.8 (2.8;5.3)	4.1 (2.8; 8.4)	3.7 (2.7; 5.7)	3.9 (2.9; 8.9)	3.8 (2.8; 4.8)	4.5 (2.8; 8.3)	3.6 (3.1; 5.0)
Cholesterol Z-score	0.2 ± 2.34	−0.9 ± 0.8	0.17 ± 2.16	−0.8 ± 1.0	−0.02 ± 1.96	−0.7 ± 1.3	0.14 ± 2.6	−0.8 ± 1.0	0.25 ± 1.97	−0.7 ± 1.0
Triglyceride mmol/L	0.99 ± 0.55		1.08 ± 0.44	0.49 ± 0.25	1.24 ± 0.71	0.70 ± 0.56	1.37 ± 0.78	0.77 ± 0.36	1.61 ± 1.08	0.87 ± 0.45
Triglyceride Z-score	1.49 ± 1.75		1.24 ± 1.06	0.0 ± 0.5	1.34 ± 1.47	0.2 ± 1.0	1.34 ± 1.37	0.2 ± 0.7	1.42 ± 1.60	0.5 ± 0.5
Testosterone nmol/L	0.38 ± 0.13	0.12 ± 0.06	1.15 ± 1.50	1.54 ± 3.1	5.43 ± 4.54	7.2 ± 3.6	9.29 ± 5.23	13.78 ± 6.25	10.37 ± 5.63	17.3 ± 5.85
Testosterone Z-score	−0.13 ± 0.38	−0.25 ± 0.75	−0.01 ± 0.69	−0.45 ± 1	−0.30 ± 1.03	0.8 ± 1.0	−0.67 ± 1.19	0.71 ± 1.02	−2.10 ± 1.78	0.31 ± 1.29
IGF-1 (µg/L)	199 ± 72		191 ± 83		338 ± 123		359 ± 117		338 ± 90	
IGF-1 Z-score	1.02 ± 1.22		−0.10 ± 1.31		0.44 ± 1.37		0.16 ± 1.30		0.19 ± 1.25	
TSH µUI/L	2.45 ± 1.08		2.46 ± 0.98		2.71 ± 1.27		2.44 ± 1.31		2.61 ± 1.48	
Free T4 µmol/L	13.96 ± 1.50		13.48 ± 2.21		13.14 ± 1.71		13.77 ± 2.00		14.02 ± 2.34	

Variables are expressed as mean ± SD or median (5th and 95th percentiles) when non-normally distributed. Abbreviations: IGF-1, insulin-like growth factor 1; TSH, thyrotropin (thyroid stimulating hormone).

Whereas height was comparable in obese and lean boys, TBLH BMC values (as absolute values, Z-scores, and height-adjusted Z-scores) were higher in obese compared to lean boys: the mean adjusted difference (on height and age) between obese and lean boys for TBLH BMC, TBLH BMC Z-score, and TBLH BMC HAZ-score was 241 ± 20 g/cm^2^, 1.14 ± 0.07 Z-score, and 1.16 ± 0.08 HAZ-score (*P* < .001 for all comparisons). Higher values were observed in obese boys for each age class (Table 1).

The mean adjusted difference (on height and age) between obese and lean boys for lumbar BMD, lumbar BMD Z-score, and lumbar BMD HAZ-score was 0.026 ± 0.009 g/cm^2^, 0.31 ± 0.09 Z-score, and 0.33 ± 0.09 HAZ-score (*P* < .01 for each comparison). By age classes, values were not significantly different between 8 and 14 years, tended to be higher in obese compared to lean boys between 14 and 16, and were higher between 16 and 18 years (Table 1).

**Figure 2.**
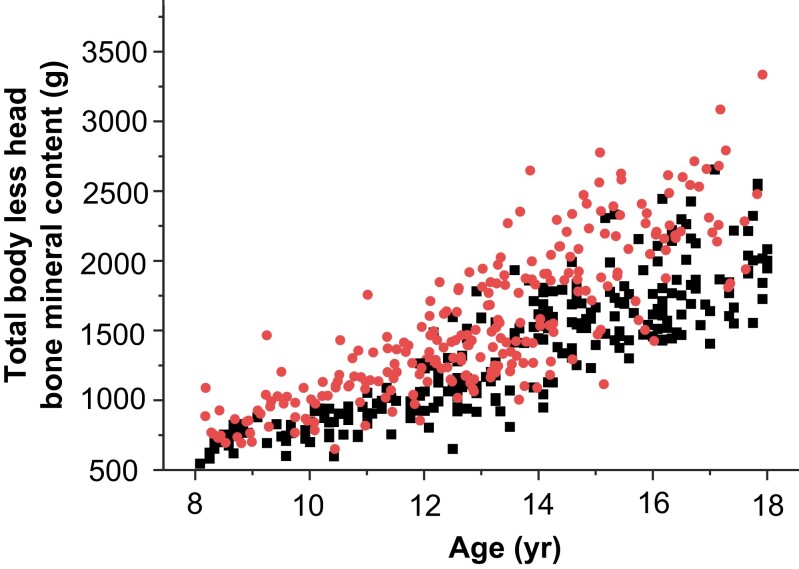
Scatterplot of total body less head bone mineral content (g) according to age (years) in overweight and obese boys (red dots) and lean boys (black squares).

These results showed an actual increase in BMC TBLH and lumbar BMD in obese boys compared to lean boys, which was not attributed to an increase in height. Figure 2 shows the dispersion of TBLH BMC according to age for obese and lean boys.

### Association of BMI, LBMI, and FMI With Bone Mineralization in Obese Compared to Lean Boys

To further characterize the effect on bone mineralization of an increase in mechanical load associated with weight in obese compared to lean boys, we studied the relationships between bone mineralization and BMI by multiple regression analyses (Table 3). We also examined the relationship between bone mineralization, lean body mass index, and fat mass index to determine how much the 2 body compartments influenced mineralization.

**Table 3.** Relationships between total body less head bone mineral content (g), lumbar bone mineral density (g/cm^2^), and body mass index (kg/m^2^) (Model 1), fat mass index (kg/m^2^), and lean body mass index (kg/m^2^) (Model 2)

	TBLH BMC (g)	*P*	Lumbar BMD (g/cm^2^)	*P*
Regression coefficient	B ± SD		B ± SD	
**Model 1**	Multiple *R^2^* 0.86	<.0001	Multiple *R^2^* 0.66	<.001
BMI (kg/m^2^) if lean	39.5 ± 6.5	<.0001	0.10 ± 0.01	<.001
BMI (kg/m^2^) if obese	24.6 ± 2.9	<.0001	0.10 ± 0.01	<.0001
**Model 2**	Multiple *R^2^* 0.89	<.0001	Multiple *R^2^* 0.69	<.0001
FMI (kg/m^2^)	−9.0 ± 3.5	.01	−0.002 ± 0.002	NS
LBMI (kg/m^2^)	78.2 ± 5.0	<.0001	0.03 ± 0.003	<.0001

All models were adjusted on height and age. Each gain of one unit of the predictor is associated with a gain of B ± SD of the dependent variable (bone mineralization).

In all models, age and height were adjusting variables.

Model 1 (TBLH BMC) There was a significant interaction between BMI and obesity status, meaning that the relationship between BMI and TBLH BMC was different between obese and lean boys (mean difference −15.0 ± 6.3 g per unit of BMI, *P* = .01)

Model 1 (lumbar BMD) There was no significant interaction between BMI and obesity status, meaning that the relationship between BMI and lumbar BMD was not different between obese and lean boys (mean difference −0.002 ± 0.003 g/cm^2^ per unit of BMI, *P* = NS). Nonsignificant interaction terms were removed from the final model.

Model 2 (TBLH BMC) There was no significant interaction between LBMI and obesity status, meaning that the relationship between LBMI and TBLH BMC was not different between obese and lean boys (mean difference −7.7 ± 7.3 g per unit of LBMI, *P* = NS). There was no interaction between FMI and obesity status (the relationship between FMI and TBLH BMC was not different between obese and lean boys), and between FMI and LBMI. All nonsignificant interaction terms were removed from the final model.

Model 2 (Lumbar BMD) There was no significant interaction between LBMI and obesity status, meaning that the relationship between LBMI and lumbar BMD was not different between obese and lean boys (mean difference −0.003 ± 0.004 g/cm^2^ per unit of LBMI, *P* = NS). There was no interaction between FMI and obesity status, and between FMI and LBMI. All nonsignificant interaction terms were removed from the final model.

Abbreviations: BMC, bone mineral content; BMD, bone mineral density; BMI, body mass index; FMI, fat mass index; LBMI, lean body mass index; TBLH, total body less head.

**Figure 3.**
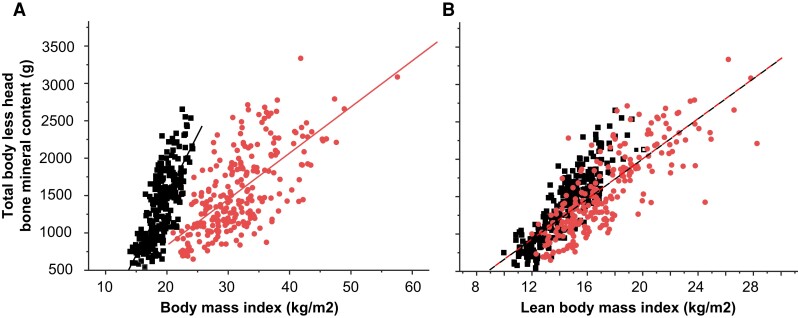
Scatterplot of total body less head bone mineral content (g) according to body mass index (A), and lean body mass index (B) in overweight and obese boys (dots) and lean boys (squares). After adjustment on height and age, the slope of the regression line was 39.5 ± 6.5 g of TBLH BMC for a gain of 1 kg/m^2^ of BMI in lean subjects, vs 24.6 ± 2.9 g of TBLH BMC for a gain of 1 kg/m^2^ of BMI in obese subjects (*P* < .05). After adjustment on height and age, the slope of the regression line was 78 ± 5 g of TBLH BMC for a gain of 1 kg/m^2^ of LBMI, with no difference between lean and obese boys. Fat mass index was negatively related to TBLH BMC (−9.0 ± 3.5 g of TBLH BMC for a gain of 1 kg/m^2^ of FMI), with no difference between lean and obese boys.

Each 1 kg/m^2^ increase in BMI was associated with an increase of +39 ± 6 g of TBLH BMC in lean subjects vs +25 ± 3 g of TBLH BMC in obese subjects (*P* < .05): the effect of a similar mechanical load (an increase of 1 kg/m^2^ in BMI) on bone mineralization was approximately 40% lower in obese compared to lean boys. More specifically, each 1 kg/m^2^ increase in lean body mass index had a similar positive effect in lean and obese boys (+78 ± 5 g of TBLH BMC, not different between obese and lean boys). In contrast, the fat mass index had a significant negative effect: each 1 kg/m^2^ increase in FMI was associated with a decrease of 9 ± 3 g of TBLH BMC. These findings suggest that the rise in TBLH BMC observed in obese boys cannot sustain the increased mechanical load associated with weight (Fig. 3).

On the opposite, the relationship between BMI and lumbar BMD and between LBMI and lumbar BMD were not influenced by the obesity status, suggesting that lumbar BMD responded similarly to the mechanical load of weight in obese and lean boys (Table 3)

### Factors Influencing Bone Mineralization

To further study the factors influencing bone mineralization, we performed path analyses for the TBLH BMC expressed as Z-score to overcome the effect of age on bone mineralization. We also performed the analyses separately in lean and obese boys since, besides DXA measurements, all other biological variables were measured using different methods between lean and obese subjects.

#### Path analyses for TBLH BMC Z-score in lean boys (Fig. 4 and Table 4)

This path analysis shows that TBLH BMC Z-score was directly influenced by LBMI and height Z-scores (positively) and indirectly influenced by testosterone, FMI, and height Z-scores (positively, mediated through LBMI Z-score).

**Figure 4.**
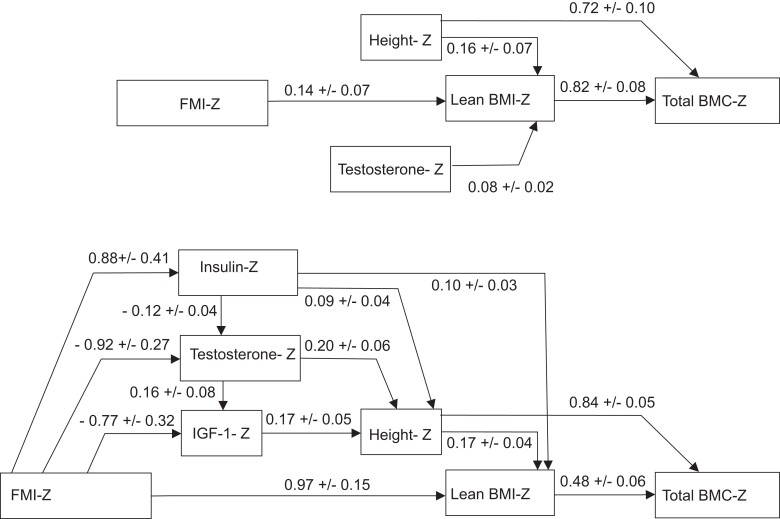
Path analyses for total body less head BMC Z-score in lean boys (A) and overweight and obese boys (B). Full line = significant path. Numbers disclosed the mean regression coefficients (B ± SD). A gain of one unit of the predictor is associated with a gain of B ± SD of the outcome variable. For direct and indirect paths details, see Tables 4 and 5. IGF-1 and thyroid hormones were not measured in lean subjects.

**Table 4.** Significant direct and indirect predictors of total body less head BMC Z-score in lean boys

Significant direct predictors of total body less head BMC Z	Estimate ± SE	*P*
LBMI Z ⇒ TBLH BMC Z	0.81 ± 0.08	<.0001
Height Z ⇒ TBLH BMC Z	0.72 ± 0.10	<.0001

Significant indirect predictors of total body less head aBMC Z	Estimate ± SE	*P*
Height Z ⇒ Lean BMI Z ⇒ TBLH BMC Z	0.13 ± 0.06	.04
FMI Z ⇒ Lean BMI Z ⇒ TBLH BMC Z	0.12 ± 0.05	.04
Testosterone Z ⇒ Lean BMI Z ⇒ TBLH BMC Z	0.06 ± 0.02	.002

χ² *P* value of the model = .90, ratio of χ² to df = 0.1; CFI = 1.0; SRMR = 0.0048; RMSEA = 0.000 (0.00; 0.05). Only significant direct and indirect predictors of the TBLH BMC Z-score were indicated. *R^2^* for TBLH BMC Z-score = 0.42 (0.32; 0.51). *R^2^* for LBMI Z-score 0.07 (0.019; 0.14). IGF-1 and thyroid hormones were not measured in lean subjects. Abbreviations: BMC, bone mineral content; BMI, body mass index; FMI, fat mass index; TBLH, total body less head.

**Table 5.** Significant direct and indirect predictors of total body less head BMC Z-score in obese boys

Significant direct predictors of TBLH BMC Z	Estimate ± SE	*P*
LBMI Z ⇒ TBLH BMC Z	0.48 ± 0.06	<.0001
Height Z ⇒ TBLH BMC Z	0.84 ± 0.05	<.0001

Significant indirect predictors of TBLH aBMC Z	Estimate ± SE	*P*
Height Z ⇒ Lean BMI Z ⇒ TBLH BMC Z	0.08 ± 0.02	.0008
Insulin ⇒ Height Z ⇒ TBLH BMC Z	0.08 ± 0.03	.01
Insulin ⇒ Height Z ⇒ Lean BMI Z ⇒ TBLH BMC Z	0.007 ± 0.003	.04
Insulin ⇒ Lean BMI Z ⇒ TBLH BMC Z	0.05 ± 0.01	.0004
FMI Z ⇒ Lean BMI Z ⇒ TBLH BMC Z	0.47 ± 0.10	<.0001
FMI Z ⇒ Testosterone Z ⇒ Height Z ⇒ TBLH BMC Z	−0.15 ± 0.06	.01
FMI Z ⇒ Testosterone Z ⇒ Height Z ⇒ Lean BMI Z ⇒ TBLH BMC Z	−0.015 ± 0.007	.04
FMI Z ⇒ IGF-1 Z ⇒ Height Z ⇒ TBLH BMC Z	−0.11 ± 0.05	.05
IGF-1 Z ⇒ Height Z ⇒ TBLH BMC Z	0.14 ± 0.04	.0007
IGF-1 Z ⇒ Height Z ⇒ Lean BMI Z ⇒ TBLH BMC Z	0.014 ± 0.005	.01
Testosterone Z ⇒ Height Z ⇒ TBLH BMC Z	0.17 ± 0.05	.0005
Testosterone Z ⇒ Height Z ⇒ Lean BMI Z ⇒ TBLH BMC Z	0.016 ± 0.006	.01

χ² *P* value of the model = .23, ratio of χ² to df = 1.3; CFI = 0.993; SRMR = 0.03713; RMSEA = 0.03758 (0.00; 0.09). Only significant direct and indirect predictors of the TBLH BMC Z-score were indicated. *R^2^* for TBLH BMC Z-score = 0.68 (0.60; 0.75). *R^2^* for LBMI Z-score 0.28 (0.18; 0.38). *R^2^* for Height Z-score 0.14 (0.06; 0.24). Thyroid hormone levels were not related to bone mineralization.

Abbreviations: BMC, bone mineral content; BMI, body mass index; FMI, fat mass index; IGF-1, insulin-like growth factor 1; LBMI, lean body mass index; TBLH, total body less head.

#### Path analyses for TBLH BMC Z-score in obese boys (Fig. 4 and Table 5)

This path analysis shows that TBLH BMC Z-score was directly influenced by LBMI and height Z-scores (positively) and indirectly influenced by insulin, height Z-scores (positively, mediated through LBMI Z-score), indirectly influenced by IGF-1, testosterone, and insulin Z-scores (positively, mediated through height and LBMI Z- scores). Finally, FMI Z-score indirectly influenced TBLH BMC Z-score both positively (through LBMI Z-score) and negatively (through its negative impact on IGF-1 and testosterone Z-scores).

#### Path analyses for lumbar BMD Z-score in obese boys

Similar results were found for path analyses for lumbar BMD Z-score (Supplementary Fig. S1) (35).

## Discussion

In this cross-sectional study in a group of 249 obese or overweight boys aged 8 to 18 years compared to 301 age and height-matched lean boys, we observed that the TBLH BMC and lumbar BMD were higher in obese compared to lean boys, even after accounting for height. These findings suggest that these higher values may be due to the increased mechanical load associated with increased weight. However, the positive relationship between body mass index and TBLH BMC by multiple regression analyses was less steep in obese boys than in lean boys, with an approximately 40% lower increase in TBLH BMC for a gain of 1 kg/m^2^ of BMI between obese and lean boys. When studying the effect of body composition and not merely BMI on bone mineralization, we found a positive relationship between lean body mass index and TBLH BMC that was similar between obese and lean boys. In contrast, fat mass index (higher in obese boys) was associated with a negative effect on TBLH BMC (after adjustment on age, height, and LBMI). These findings indicate that the increase in TBLH BMC observed in obese compared to lean boys may not be sufficient to support the increased mechanical load of obesity. In addition, path analyses, used to better understand the relationships between bone mass, lean mass, fat mass, and hormones, confirmed that lean body mass directly and significantly influenced bone mineralization in lean and obese boys. Lean body mass was positively modulated by several other factors in obese boys: height, IGF-1, testosterone, and insulin (indirect modulators of bone mineralization). The influence of fat mass on bone mineralization was only indirect and dual: positive through lean body mass and insulin and negative through its negative impact on IGF-1 and testosterone. Our study indicates a complex interplay between fat mass, lean mass, and hormonal factors. These findings may explain why the increase in mechanical load associated with increased lean and fat mass may not translate into a sufficient response of bone mineralization.

By increasing mechanical stress, overweight promotes osteoblastic activation and bone formation, and inhibits osteoclastogenesis and bone resorption (13, 14). Our results complete this postulate: lean mass was strongly associated with bone mineralization, whereas fat mass was negatively (and more weakly) associated with bone mineralization (TBLH BMC, but not lumbar BMD, which was not related to FMI). The negative effect of fat mass (after adjustment on lean body mass) agrees with several studies: meta-analyses performed mostly in adults showed that fat mass percentage generally correlated negatively with bone mineralization but not absolute fat mass (36, 37). At least, this showed that only the lean body mass component of the BMI had a positive impact on bone mineralization. Lumbar BMD did not differ between obese and lean boys between 8 and 14 years of age, but it did differ for older ages. This may be due to a lack of power, as the trend toward higher mean lumbar BMD values in obese boys was observed from age 10 onwards (see Table 1). It is also possible that the magnitude of the difference increased with age and pubertal stage, suggesting that sex steroids potentiated the change in lumbar BMD associated with increased weight. Notably, the effect of testosterone (and estrogen) changes on bone density in adults is known to be more marked at the lumbar level than at other sites (38). An age-specific effect of obesity on trabecular architecture may also be present: whether the trabecular bone score can help characterize bone health in obese children has yet to be validated (39).

Path analyses were used to describe dependencies among bone mass, lean mass, fat mass, and hormones. They confirmed that lean mass was positively associated with bone mass in obese and lean subjects (as with multiple regression analyses). In obese boys, they showed that insulin, testosterone, and IGF-1 were positively and indirectly associated with bone mass (through their positive effects on height and lean mass). Finally, they showed that fat mass had both (i) a direct positive effect on lean mass (an already well known association in obese subjects) (40), and therefore an indirect positive effect (through lean mass) on bone mass; and (ii) a direct negative effect on IGF-1 and testosterone (see below for discussion), and therefore an indirect negative effect on bone mass. This dual effect of fat mass may explain why the influence of fat mass on bone mineralization is sometimes negative, sometimes neutral depending on studies, whereas the influence of lean mass is consistently found positive (36, 37). The increase in the number of fractures in obese children could relate to the relative fragility of their bones regarding the weight stress (37, 41, 42):

IGF-1 is known to increase bone formation (19, 43). A recent Mendelian randomization study in adults supported a role for IGF-1 in preventing fracture, possibly and partly mediated by greater bone mineral density (44). Our study brings new information and suggests an indirect role of IGF-1 on bone mineralization, mainly mediated through LBMI and height. Of note, age-related IGF-1 values were negatively affected by fat mass Z-scores, in agreement with some, but not all, studies in children and adolescents (24, 45).

Concerning the role of insulin resistance, we studied the effect of insulin levels on bone mineralization. We showed that fasting insulin was positively associated with LBMI and, therefore, indirectly associated with bone mineralization. The impact of hyperinsulinism on bone has been studied mainly in the adult population with type 2 diabetes (46), with the possible confounding effect of hyperglycemia. Experimental studies, as well as studies in murine models, suggest that hyperinsulinemia without hyperglycemia leads to a significantly higher trabecular and cortical bone mass, likely through a reduced bone turnover (19, 47).

We found that levels of testosterone indirectly positively affected bone mineralization through its effect on lean mass. These findings agree with the positive impact of androgens on bone health, which has been demonstrated in human and animal studies (19, 48). Of note, obesity has been associated with decreased age-adjusted testosterone values in adolescents (23, 49). In our research, path analyses showed that the FMI Z-score had a negative impact on the testosterone Z-score and, therefore, an indirect negative impact on bone mineralization.

Our study has several limitations. It was cross-sectional and retrospective, and we could not confidently retrieve data on lifestyle, diet, calcium intake, physical activity, and vitamin D levels, which have been showed to impact bone mineralization in some (18), but not all, clinical studies (50, 51). However, the age-related change in height and testosterone levels observed in the obese children from our study has also been observed in other studies, thus suggesting that our cohort resembles published cohorts, and that recruitment bias was limited (8, 49). Another limitation in the analyses of the effect of sex steroids on bone mineralization is that we did not measure free testosterone and estrogen levels and, therefore, could not study their impact (19, 52, 53). This study is the first to evaluate hormonal factors in such a large group of obese or overweight boys and to understand their effects on bone mineralization.

In conclusion, we have shown that bone mineralization in obese children and adolescents, although increased in response to the increased mechanical load of weight, did not follow the same positive relationship with BMI as that observed in lean boys. Lean body mass, however, had a similar positive relationship with bone mineralization in obese and lean boys, whereas fat mass had a negative impact. Insulin, testosterone, and IGF-1 were all related to lean body mass index and indirectly influenced bone mineralization.

## Data Availability

We will consider sharing data collected for the study, including deidentified individual participant data that underlie the results reported in this article, on receipt of a request detailing the study hypothesis and statistical analysis plan. All requests should be sent to the corresponding author. The corresponding author will discuss all requests and make decisions about whether data sharing is appropriate based on the scientific rigor of the proposal. All applicants will be asked to sign a data access agreement.

## Abbreviations

BMC: bone mineral content
BMD: bone mineral density
BMI: body mass index
CDC: Centers for Disease Control
DXA: dual-energy x-ray absorptiometry
FMI: fat mass index
HAZ: height-adjusted Z-score
IGF-1: insulin-like growth factor 1
LBMI: lean body mass index
NHANES: US National Health and Nutrition Examination Survey
RMSEA: root mean square error of approximation
SRMR: standardized root mean squared residual
T4: thyroxine
TBLH: total body less head
TSH: thyrotropin (thyroid stimulating hormone)
